# A Systematic Review of the Prospective Relationship between Child Maltreatment and Chronic Pain

**DOI:** 10.3390/children8090806

**Published:** 2021-09-15

**Authors:** Teresa J. Marin, Rebecca E. Lewinson, Jill A. Hayden, Quenby Mahood, Meghan A. Rossi, Brittany Rosenbloom, Joel Katz

**Affiliations:** 1 Department of Psychology, Faculty of Health, York University, Toronto, ON M3J 1P3, Canada; lewinson@yorku.ca (R.E.L.); rosenbloom.brittany@gmail.com (B.R.); jkatz@yorku.ca (J.K.); 2 Department of Community Health and Epidemiology, Faculty of Medicine, Dalhousie University, Halifax, NS B3H 4R2, Canada; jhayden@dal.ca; 3 SickKids Hospital, Toronto, ON M5G 1X8, Canada; quenby.mahood@gmail.com; 4 Department of Psychology and Neuroscience, Faculty of Science, Dalhousie University, Halifax, NS B3H 4J1, Canada; meghan.audrey.rossi@gmail.com

**Keywords:** child maltreatment, chronic pain, systematic review, PTSD

## Abstract

Objective: The present systematic review aimed to evaluate the association between childhood maltreatment and chronic pain, with specific attention to the temporal nature of the relationship and putative moderators, including, the nature (type), timing of occurrence, and magnitude of maltreatment; whether physical harm or injury occurred; and whether post-traumatic stress disorder (PTSD) subsequently developed. Method: We included studies that measured the prospective relationship between child maltreatment and pain. Medline, EMBASE, PsycINFO, and CINAHL were searched electronically up to 28 July 2019. We used accepted methodological procedures common to prognosis studies and preregistered our review (PROSPERO record ID 142169) as per Cochrane review recommendations. Results: Nine studies (17,340 participants) were included in the present review. Baseline participant age ranged from 2 years to more than 65 years. Follow-up intervals ranged from one year to 16 years. Of the nine studies included, three were deemed to have a high risk of bias. With the exception of one meta-analysis of three studies, results were combined using narrative synthesis. Results showed low to very low quality and conflicting evidence across the various types of maltreatment, with the higher quality studies pointing to the absence of direct (non-moderated and non-mediated) associations between maltreatment and pain. PTSD was revealed to be a potential mediator and/or moderator. Evidence was not found for other proposed moderators. Conclusions: Overall, there is an absence of evidence from high quality studies of an association between maltreatment and pain. Our results are limited by the small number of studies reporting the relationship between child maltreatment and pain using a prospective design. High quality studies, including prospective cohort studies and those that assess and report on the moderators described above, are needed to advance the literature.

## 1. Introduction

Child maltreatment is common, with at least one in seven children having experienced abuse and/or neglect in the past year [1]. These experiences are traumatic to the individual and have serious life-long consequences, including depression, anxiety, PTSD, and suicide [2,3,4,5,6]. In addition to the psychological and emotional toll, research studies have reported that child maltreatment co-occurs with or predicts impaired physical health [7], including increased risk of chronic pain [7,8]. Chronic pain is often longstanding, with an average duration of seven years [9], resulting in a tremendous burden on individuals, their families and society at large [10].

Despite these findings, other studies have failed to show a relationship between child maltreatment and pain [11], and even for those that have, there are a number of methodological concerns that limit these findings. Specifically, most studies have relied on retrospective designs in combination with self-report measures of child maltreatment; thus, a positive association between maltreatment and pain may indicate a causal relationship, but it is also plausible that a third variable, such as a reporting bias, is driving the effect. The concern here is not only that people with adult health problems may over report childhood adversity, but that individuals who are free of pain tend to under-report these events [12], thereby creating a spurious association between maltreatment and pain [13]. We must, therefore, utilize studies that evaluate, in a prospective manner, the relationship between maltreatment and pain, including the use of substantiated measures of child maltreatment, to delineate the nature of this association [11,14].

In addition to these methodological issues, growing evidence from the fields of mental health and pediatric pain suggest that the association between maltreatment and pain may hinge on key moderating factors which could explain variability in findings across studies. These factors include (1) the presence of post-traumatic stress disorder (PTSD) or PTSD symptoms (PTSS) [15,16,17,18], (2) the particular type of maltreatment and whether it involved physical harm [8,13,16,19], (3) its frequency and stability over time [20,21,22], (4) and whether it occurred within a critical developmental window [23,24]. The following sections present evidence for each of these potential moderators.

### 1.1. PTSD

Among children and adolescents who are exposed to maltreatment, their psychological response to maltreatment, including the emergence of PTSD or PTSS, may have consequences for chronic pain. Indeed, individuals with PTSD are at increased risk of developing chronic pain [25]. It has been proposed that the symptoms of these two conditions are mutually maintaining, such that the affective, physiological, and behavioural symptoms of PTSD maintain and/or exacerbate the pain experience, and that pain itself maintains and/or intensifies PTSS [15,16,18]. This relationship has also been shown in younger populations: compared to youth who do not report chronic pain, those who do show greater increases in symptoms of PTSD [17]. Accordingly, if a child or adolescent exposed to a trauma develops PTSD or partial PTSD, they may be at greater risk of chronic pain. Raphael and Widom [26] examined the relationships between childhood maltreatment (verified by criminal court records), PTSD, and pain 30 years later. They found that participants who had a history of PTSD and childhood abuse were at significantly greater risk of developing pain in adulthood. Clearly, an in-depth look at the role of PTSD across studies will clarify our understanding of the trauma–pain relationship.

### 1.2. Type of Child Maltreatment

Another feature of child maltreatment that may impact its association with chronic pain is the particular type of abuse, including physical abuse, sexual abuse, emotional abuse, neglect, and exposure to intimate partner violence. Although evidence from the mental health literature points to equivalence across abuse types [27,28], less is known about the effects of abuse type as they relate to the child maltreatment and chronic pain association. Results from cross-sectional studies are mixed. For example, Scott and colleagues [29] showed equivalence across abuse types in relation to pain outcomes, while Stickley and colleagues [19] showed some evidence of specificity in a study of adult-onset chronic pain conducted in Japan. In particular, sexual and physical abuse were associated with chronic pain, whereas family violence and neglect failed to show independent effects.

In addition to accounting for the specific type of abuse, we propose that even greater specificity is needed at the level of physical harm or injury. Indeed, cases of sexual and physical abuse have a physical component in addition to a psychological component, which means that they may have a direct link to pain [13,16]. Moreover, traumatic events involving physical injury are more likely to lead to PTSD compared to events without a physical component [30], and PTSD is highly co-morbid with chronic pain [25]. Therefore, we expect that child maltreatment resulting in physical harm or injury will be associated with a higher risk of chronic pain. Although the role of physical harm, resulting from violence, has received little attention in the child maltreatment literature, studies of sexual abuse and rape provide indirect evidence that physical harm may be an important factor. This is exemplified by a meta-analysis by Paras et al. [8], who reported an association between sexual abuse and somatic symptoms, including fibromyalgia and pelvic pain, but only when limiting the type of sexual abuse to rape. However, it is unclear whether this effect is driven by the physical harm associated with rape or if it is mediated by psychological factors. Indeed, research evidence shows that sexual abuse, including rape, is more predictive of PTSD compared to sexual abuse without rape [31]. Clearly, it is very challenging (and perhaps impossible) to disentangle the psychological and physical components of sexual abuse and rape. However, this work demonstrates the importance of specifying the nature of the maltreatment and considering the role of physical violence and resulting injury in the maltreatment–pain relationship.

Even if physical harm is instrumental in affecting the maltreatment–chronic pain relationship, clearly other factors are involved. For example, some people develop chronic pain following non-injurious forms of trauma, such as children who are exposed to domestic partner violence [32]. Moreover, studies of traumatic injury show only a weak link between injury severity and chronic pain [33], highlighting the importance of emotional and psychological variables. Furthermore, this literature is complicated by the high comorbidity between various types of maltreatment [20], making it challenging to differentiate the impact of maltreatment types on pain outcomes or to parse out the independent effects of physical versus emotional trauma [13]. Finally, studies of child maltreatment and later health often fail to account for the role of non-inflicted injury and intentional self-injury, both of which occur more frequently among maltreated children [32,34,35]. Indeed, there are likely to be multiple pathways from child maltreatment to the initiation and persistence of pain. Some pathways may be specific to the type of maltreatment and others may apply across maltreatment types.

### 1.3. Frequency and Chronicity of Child Maltreatment

Another feature of child maltreatment that may impact its association with chronic pain is its intensity, captured by (1) the frequency of discrete episodes of abuse and (2) its chronicity over time. Much of what is known about the frequency of early life exposures and health outcomes comes from studies of adverse childhood experiences (ACEs). Typical ACEs include child sexual abuse, child physical abuse, neglect, and childhood exposure to domestic abuse, parental psychopathology and early parental loss. This work points to a graded association between the number of adverse exposures and physical and mental health outcomes in adulthood [36,37,38], and mounting evidence from cross-sectional studies suggests a similar pattern of findings for pain outcomes [19,29,39,40]. Indeed, a recent study reported that the number of ACEs was a better predictor than the specific type of trauma of dysmenorrhea, chronic headache, and chronic back pain [22]. Although less is known about whether repeated incidents of maltreatment have a cumulative impact on chronic pain development, results from one prospective study showed that more documented exposures to maltreatment (i.e., higher numbers of incidents) predicted more severe health and behavioral outcomes (e.g., substance use and mental health treatment) among children and adults [41], thereby mirroring the findings from the ACEs studies. We, therefore, expect to find a similar pattern in regard to chronic pain outcomes, with more frequent maltreatment episodes incurring greater risk.

That said, a singular focus on the frequency of maltreatment episodes may leave out important information about its overall intensity (which is captured by the combination of frequency and chronicity/duration). As articulated by Gilbert and colleagues [20], for some youth, maltreatment is more accurately conceptualized as a chronic condition, than as a single event (or a series of events). In this regard, it is also important to capture the chronicity of maltreatment over time, although to our knowledge there is very little evidence examining the health consequences of chronic versus shorter-term maltreatment. However, we propose that this dimension may be especially relevant in the case of childhood neglect. Child neglect is characterized by lack of parental care and nurturance, and as such, often involves chronic situations that are not as easily identified as specific incidents, as are specific instances of physical abuse of sexual abuse [42]. The health consequences of these chronic family situations have been captured by research on risky families [21]. Risky family environments, defined by high conflict, deficient nurturing, and neglect, are related to a greater risk of chronic illnesses, mental health conditions, and early mortality across the lifespan. Therefore, we would expect the chronicity of neglect and other forms of maltreatment to relate to the subsequent presence of chronic pain.

### 1.4. Developmental Stage of Maltreatment

In addition to the frequency and stability of maltreatment, the developmental stage during which it occurs may have implications for later chronic pain. Developmental timing is related to chronicity, as maltreatment that occurs across a longer timespan will inevitably cross more developmental stages. However, exposure to maltreatment, even when it is short-lived, may have long-term effects if it occurs within a critical developmental window. In this regard, attention has focused mostly on early life models. For example, Kaplow and Widom [43] showed that early onset childhood maltreatment (i.e., early (0–5 years of age) versus later (6–11 years of age)) predicted more severe symptoms of depression and symptoms of anxiety when the participants were adults. Similarly, research evidence indicates that risk for depression and thoughts of suicide are influenced by the developmental age when first exposed to child maltreatment: exposure during early childhood (0–5 years) is more detrimental than exposure during adolescence [23]. First exposure to child maltreatment between 0–5 years of age (early childhood) has also been linked to elevated risk for PTSD relative to first exposure during middle childhood or adolescence [24], a finding which may have implications for chronic pain [17,25]. Taken together, the developmental timing of maltreatment appears to be an important factor in determining chronic pain outcomes among maltreated children.

### 1.5. The Present Study

We conducted a systematic review of the relationship between child maltreatment and chronic pain selecting for studies that used a prospective design and focusing on factors that are likely to shape this relationship. We aimed to answer four research questions:

1. Does the literature show a temporal relationship between child maltreatment and chronic pain?
2. Do individuals who are exposed to child maltreatment and develop PTSD or partial PTSD differ in chronic pain outcomes from those who are exposed but do not develop these symptoms?
3. Does the particular type of maltreatment influence pain outcomes? In answering this question, we sought to evaluate the differential association among sexual abuse, physical abuse, emotional abuse, and neglect. For sexual and physical abuse, we also examined whether the presence of abuse-related physical harm influenced the relationship between abuse and chronic pain.
4. Does the intensity of maltreatment predict chronic pain? To answer this question, we examined the relationships between the frequency (i.e., has it occurred 10 times or more?) and chronicity (i.e., has it been persisting for at least six months?) of maltreatment and subsequent chronic pain.
5. Does the developmental stage (early childhood, middle childhood, or adolescence) during which maltreatment occurred predict chronic pain outcomes?

## 2. Materials and Methods

The present systematic review was preregistered with PROSPERO (https://www.crd.york.ac.uk/prospero/display_record.php?ID=CRD42019142169.44 (accessed on 9 September 2021)) [44].

### 2.1. Inclusion Criteria

Studies were included if they investigated maltreatment occurring in youth (i.e., 18 years or younger) and later experience of pain. We included two types of study designs: (1) Prospective cohort studies with a clearly defined measure of child maltreatment (retrospective self-report or verified maltreatment) and a measure of pain obtained at least three months later (in childhood, adolescence or adulthood depending on age when first recruited); (2) cross-sectional studies with retrospective verified reports of child maltreatment (e.g., court documentation) and a measure of pain occurring at least three months later. We included only peer-reviewed articles.

### 2.2. Exclusion Criteria

We excluded (1) studies of experimental, lab-based pain, (2) clinical trials, (3) case reports, review articles, book chapters, theses, letters to the editor, commentaries and editorials, qualitative studies and meeting abstracts, and (4) articles written in languages other than English.

### 2.3. Defining Child Maltreatment

Child maltreatment and neglect include acts of commission (e.g., physical abuse) or omission (e.g., neglect) by a parent or other caregiver resulting in harm, potential for harm, or threat of harm to the child’s health, survival, development, or dignity [45]. The five most common forms of maltreatment are sexual abuse, physical abuse, emotional maltreatment and exposure to domestic violence and neglect [45,46]. We included studies that assessed child maltreatment by self-report questionnaires and interviews, reports by parents and other caregivers, and information extracted from official documents, such as court documentation of child maltreatment and medical documentation of abuse-related physical trauma.

Specific features of the maltreatment exposure were coded. These included the type, frequency, chronicity, and developmental timing of the exposure. See Table S1 for operational definitions used.

### 2.4. Defining PTSD

To examine the moderating role of PTSD, we included studies that report diagnosed PTSD, as assessed by clinical interviews, such as the National Institutes of Mental Health Diagnostic Interview Schedule [47], as well as subsyndromal PTSD (i.e., symptoms typically measured via self-report questionnaires, such as the PTSD Checklist) [48].

### 2.5. Defining Pain Outcomes

The primary outcome measure was chronic pain, which we defined as a duration of pain greater than three months [49]. Studies were included if they measured pain intensity or pain frequency, and if they reported a chronic pain condition categorically (e.g., presence of chronic abdominal pain, presence of headaches). Studies were included if they measured pain by self-report instruments (e.g., visual analog scale, verbal rating scale, numeric rating scale, McGill Pain Questionnaire) [50], clinician interviews, reports from parents, or from clinical examinations.

We included studies that measured pain more than three months after the trauma. This was done to avoid capturing pain that may have been present as a direct result of maltreatment (i.e., acute pain). That said, many studies reporting pain outcomes do not indicate the duration of the pain condition, making it uncertain whether the pain is chronic. Because of this uncertainty, we made a decision, a priori, to include studies regardless of whether pain duration was reported and, if possible, to account for measurement issues of this type when we analyzed the results.

Regarding secondary outcomes, studies were included if the reported pain-related outcomes (e.g., pain disability or pain interference). Whenever available, information about pain medication use was collected.

### 2.6. Search and Screening Strategy

An experienced librarian (QM) performed electronic searches of Medline, EMBASE, PsycINFO, and CINAHL. We adapted the search strategy from a published systematic review on risk factors for chronic pain [51]. Search terms initially covered 3 general sets: (1) Exposure: trauma, (2) Outcome: chronic pain, and (3) Study design: comprised of search terms including “risk” and “association” (see Medline terms we searched reported in Table S2). We customized the electronic searches for each database, using both free text and index terms. We screened the citations for relevance using title/abstract and full-text review to identify studies to include in the present review. Following the full-text review, a decision was made to conduct a more focused review on child maltreatment and chronic pain. The literature search was then revised to reflect this new focus, and this more specific search strategy was used to update the search prior to finalizing the manuscript.

Given the limitations associated with solely searching electronic databases [52], we also combed through the literature cited from earlier published reviews of child maltreatment and pain [8,11,13,14,53,54]. Finally, we reviewed the reference sections of the studies we included and we conducted citation searches of four influential publications [19,20,29,55].

### 2.7. Data Extraction

Pairs of three reviewers (T.M., R.L., B.R.) extracted data onto electronic extraction forms and later met to discuss the data and reach consensus. The three reviewers consulted J.K., a fourth reviewer, to reach consensus through discussion when pairs of reviewers disagreed. See Table S3 for a record of variables we extracted.

### 2.8. Assessing Risk of Bias

Each study was assessed for risk of bias using the Quality in Prognosis Studies (QUIPS) tool [56]. Six domains were assessed: participation, attrition, risk factor measurement, outcome measurement, confounding, and data analysis and reporting. To inform the risk of bias judgment, we considered responses to the prompting items for each of the six domains. For the confounding domain, risk of bias was determined by taking into account the extent to which data analyses were adjusted for possible confounders using the following three categories of adjustment: (1) unadjusted (i.e., analyses conducted without covariates or confounders in the model; (2) minimally adjusted (i.e., analysis using sex and age of participants as covariates); (3) adequately adjusted (i.e., analysis using the following covariates: sex; age; baseline pain; social status defined by family income, parent education or a similar variable; negative affect including a measure of depression or anxiety or neuroticism; and the presence of adult abuse or current abusive relationship). For analyses examining the association of a specific type of abuse on pain outcomes, we considered the model to be adequately adjusted if it also controlled for other types of abuse. Unadjusted studies received a high risk of bias rating. Minimally adjusted studies received a moderate risk of bias rating. Adequately adjusted studies received a low risk of bias rating. Determination of overall study validity was accomplished by classifying studies as low risk of bias when half or more than half of bias domains were rated to be low risk and serious sources of potential bias across the domains were not present. This assessment was conducted separately by T.M. and J.K., and after subsequent comparison of ratings, any lack of agreement was settled by the two assessors jointly reviewing and discussing the study in question.

### 2.9. Measures of Association Extracted

We used Hayden and colleagues’ [57,58,59] recommended methods to extract unadjusted and adjusted measures of the relationship between child maltreatment and pain. We calculated odds ratios (ORs) in the natural log scale to measure the association between child maltreatment and pain. We transformed effect sizes to the natural log scale and computed standard errors (SEs) using an appropriate formula involving a log-transformation of confidence intervals. We transformed standardized regression coefficients for continuous outcome measures to natural log odds ratios [58,59].

### 2.10. Data Synthesis

Meta-analysis was performed when ≥3 similar studies assessed the association between chronic pain and child maltreatment or one of the proposed moderator variables we outlined in the introduction. Review Manager (RevMan version 5.3, Cochrane Collaboration) was used to conduct meta-analyses. Random-effects generic inverse variance meta-analysis model was used to account for heterogeneity between studies in the exposure variable effect. Summary statistics for the meta-analysis included the pooled estimate (mean exposure effect) and the associated 95% CI. Analyses were performed once using an unadjusted model and again using an adjusted model controlling for potential confounders.

Proposed moderation models were examined using analysis of subgroups to explore between-study differences in the presence of PTSD or PTSS, maltreatment features, such as child maltreatment type, injury to the body, and the frequency, chronicity and developmental timing of the maltreatment exposure. We intended to use subgroup analysis to evaluate the potential impact of differences in the timing of outcome measurement using subgroup analysis. In particular, we were interested in exploring whether the assessment was performed in childhood/adolescence (18 years or younger) or later in life (>18 years).

Sensitivity analysis was planned to test the relationship of other variables on the association between maltreatment and chronic pain. Specifically, we intended to evaluate the effects of chronic pain measurement, risk of bias, and confounder adjustment by limiting the analyses to studies that (1) measured chronic pain in a valid manner, (2) were rated low risk of bias, and (3) adjusted for confounding factors in an adequate manner.

### 2.11. Interpretation of Results

For binary factors, we used small (OR < 1.5), moderate (1.5 ≤ OR ≤ 2), or large (OR > 2) effect sizes to define the magnitude of the observed associations [57,60]. Moderate or large effect sizes (OR ≥ 1.5) were deemed to be clinically relevant. Between-study statistical heterogeneity was considered substantial when I2 was >50%. Study results were presented qualitatively when it was deemed inappropriate to conduct a meta-analysis because of a small number (<3) of sufficiently homogeneous studies.

We evaluated overall quality of evidence of the relationship between child maltreatment and pain with a modified approach to the GRADE framework [57,61]. Internal validity, effect size and effect precision, heterogeneity, generalizability, and potential reporting bias were considered when rating the overall evidence for each study as high, moderate, low or very low.

## 3. Results

### 3.1. Description of Studies

#### 3.1.1. Results of the Search

The search of the literature identified 18,730 unique citations. After reviewing the titles and abstracts, we retrieved 476 full-text articles (456 from general trauma search, 17 from focused search update, and 3 articles identified through other sources) for further assessment and study selection. Ultimately, nine studies met our inclusion criteria. The search was last updated on 28 July 2019. See Figure S1 for the study flow diagram and Table S4 for a description of included studies.

#### 3.1.2. Included Studies

We included nine studies (17,340 participants) that examined the relationship between child maltreatment and pain [26,32,62,63,64,65,66,67,68]. Six studies took place in the United States of America [26,62,63,64,67,68], two studies were conducted in Europe [32,66], and one in Canada [65]. Four studies were cohort studies with retrospective self-reports of maltreatment [63,65,66,68], three studies followed specialized cohorts (i.e., individuals with a history of child maltreatment) and matched cohorts [26,62,69], and two were cross-sectional with retrospective verified reports of child maltreatment [32,64]. One study followed participants with a diagnosis of borderline personality disorder [63], and two followed participants who were free of pain at baseline [65,66]. Follow up periods for the longitudinal studies ranged from one year to 16 years. Two studies evaluated the relationship between child maltreatment and childhood pain [32,69], whereas the other studies evaluated the association between child maltreatment and pain in adulthood. Table S4 shows a summary of study variables and characteristics.

#### 3.1.3. Excluded Studies

We excluded 466 articles after full-text screening. The most common reasons for exclusion were: (1) no measure of child maltreatment, (2) no measure of pain or pain was not included as an outcome in the maltreatment analyses and (3) child maltreatment and pain measured cross-sectionally (with no verified reports available).

#### 3.1.4. The Measurement of Child Maltreatment

Studies in this review identified child maltreatment using various methods. Four studies relied on either self-report questionnaires [65,66] or interviews [63,68]. Three studies abstracted information from official reports [26,62,64]. One study recruited a sample of children referred for intervention due to exposure to domestic partner violence and then used parent reports and official reports to ascertain the presence of additional exposures to abuse [32]. Finally, Rimsza and colleagues [69] recruited children who had been identified as victims of sexual abuse following evaluation by a sexual abuse team at a medical centre in Phoenix, Arizona.

#### 3.1.5. The Measurement of PTSD and PTSS

Three studies measured the presence of PTSD using structured clinical interviews [26,63,68]. In addition, Beal and colleagues [62] measured baseline PTSS using the Comprehensive Trauma Interview [70].

#### 3.1.6. The Measurement of Pain

Seven studies measured pain in adulthood as the primary outcome [26,62,63,64,65,66,68], and two studies measured pain in childhood as the primary outcome [32,69]. Moreover, two studies focused on change in pain or pain status over time [65,66]. Three studies used measures of pain or pain interference that captured the experience of chronic pain for pain interference [32,64,65].

### 3.2. Risk of Bias

Six studies had an overall risk of bias that was rated to be low [26,62,64,65,66,68]. Table S5 contains a summary of the risk of bias assessments. It is notable that, although these six studies were rated as higher quality than the other studies, they each nonetheless showed moderate risk of bias due to study confounding. Indeed, none of the studies met our criteria for adequate adjustment (i.e., statistical control for age, sex, initial pain, social status, negative affect, and adult or current abuse, and for analyses examining the relationship between specific abuse types and pain, other types of abuse).

Three studies were rated to have high risk of bias overall [32,63,69]. These studies were documented to have high risk of bias in one domain (statistical analysis and reporting) [63], two domains (statistical analysis and reporting and study confounding) [32], or three domains (statistical analysis and reporting, study attrition, and outcome measurement) [69]. For each of these studies there were concerns about selective reporting, indicating that positive associations may be over-represented in the results.

### 3.3. Findings

Zero to five studies yielded similar enough data for each of our research questions, and zero to three studies were available for each of the planned meta-analyses. It was not possible to perform the intended subgroup analysis and sensitivity analysis given the small number of studies. Taken together, the level of evidence was determined to be low to very low quality. See Table S6 for a summary of the evidence for each research question and the corresponding GRADE analysis.

#### 3.3.1. Is Exposure to Any Child Maltreatment Associated with Pain at Follow-Up?

Three studies yielded low quality evidence [26,62,63] from a total of 1421 participants). These studies examined the relationship between any child maltreatment (i.e., two or more types of maltreatment measured and then combined in a single index) and pain at follow-up [26,62,63]. In unadjusted analyses, Beal and colleagues [62] found that female participants with a documented history of maltreatment were more likely to report experiencing pain at follow-up compared to women without a maltreatment history (*p* < 0.05). Biskin and colleagues [63] showed that the severity of any verbal or physical abuse was significantly and positively related to pain at follow-up (*p* = 0.001). However, adjusted analyses yielded mixed findings. In the Beal et al. study [62], after adjusting for covariates, including PTSD, there was no direct association between maltreatment status and pain (reported as not significant, “ns”). Similarly, in adjusted analyses, Raphael and Widom [26] did not find a difference in pain (ns) among participants with a history of any maltreatment (documented by court records) compared to controls (matched on demographic variables). In contrast, Biskin and colleagues [63] found that the severity of verbal and/or physical abuse was related to pain even after controlling for covariates, including other types of abuse (*p* < 0.05).

In regard to secondary outcomes, adjusted analyses reported by Raphael and Widom [26] did not reveal significant differences in pain interference when participants who had a history of any maltreatment were compared to those who did not (ns). Although Biskin and colleagues [63] measured pain interference, the relationship between any maltreatment and interference was not reported.

#### 3.3.2. Does the Presence of PTSD or PTSS Influence the Maltreatment–Pain Relationship?

One study [26] with 807 participants, which tested the moderation of the maltreatment–pain relationship by PTSD, yielded low quality evidence. Adjusted analyses revealed a significant interaction between maltreatment status and PTSD, such that maltreatment history plus lifetime PTSD was associated with elevated pain in middle adulthood compared to no history of maltreatment (*p* < 0.001). The authors state that after controlling for this effect, neither the child maltreatment alone nor PTSD alone approached statistical significance. However, the results of the statistical analysis for moderation are not fully reported, and there is no graph of the interaction effect, so there is no way to assess if the authors’ interpretation accurately reflect the data analyses.

In regard to secondary outcomes, Raphael and Widom [26] showed the same interaction effect between maltreatment status and PTSD in the prediction of pain interference (*p* < 0.001).

#### 3.3.3. Does the Type of Maltreatment Influence the Maltreatment–Pain Relationship?

Each study in the review specified maltreatment type, including the studies by Raphael and Widom [26] and Beal and colleagues [62], which also reported the overall effects of any documented history of maltreatment (as summarized above). The Raphael and Widom26 findings for each maltreatment type (sexual abuse, physical abuse, and neglect) are described in the sections below. In contrast, Beal and colleagues [62] did not report associations between each maltreatment type and pain. However, they showed results of unadjusted analyses, showing that pain did not differ significantly among women who had reported neglect only, sexual and/or physical abuse only, and both abuse and neglect. This result suggests that the effect of maltreatment on pain is uniform across maltreatment types.

##### Sexual Abuse

Very low-quality evidence from five studies (6203 participants) provided information about the longitudinal relationship between child sexual abuse and pain. Two studies with high risk of bias reported unadjusted results only [63,67]. These studies yielded mixed findings, with one study pointing to elevated pain at follow-up in survivors of sexual abuse compared to controls (*p*-value not reported) [69] and the other study showing no association (ns) [63]. It should be noted that the Rimsza et al. [69] finding is for abdominal pain, whereas findings for other pain areas (i.e., headaches, chest pain, back pain, and vaginal pain) were not reported. In addition, findings from three low risk of bias studies that adjusted for potential confounders were also mixed [26,66,71]. As reported by Linton et al. [66], history of childhood sexual abuse was unrelated to pain at follow-up, among both participants who were free of pain at baseline and those who reported pain at baseline (ns). Similarly, Raphael and Widom [26] showed no differences in pain at follow-up in cases of documented sexual abuse compared to controls (ns) [26]. In contrast, Sachs-Ericsson et al. [68] reported a statistically significant and positive association between sexual abuse and pain at follow-up (*p* < 0.001). Regarding secondary outcomes, evidence from two studies [26,66] did not show a significant relationship between childhood sexual abuse and subsequent pain interference when follow-up was completed (ns).

##### Physical Abuse

**Figure 1:**
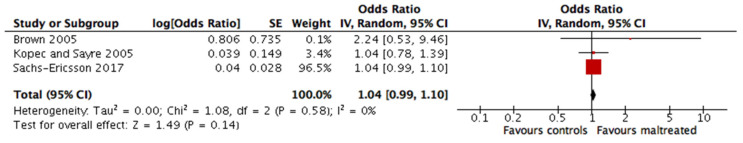
Forest plot illustrating the results of a meta-analysis (random-effects) of prospective, longitudinal studies examining the relationship between childhood physical maltreatment and pain at follow-up, after adjusting for potential confounding variables.

There was low quality evidence from four studies (15,150 participants) on the longitudinal association between child physical abuse and pain [26,64,65,68]. Each of these studies reported results that were adjusted for potential confounders. Not one study showed a statistically significant relationship between physical abuse and pain, as shown in meta-analysis of three studies with usable data (OR [95% CI] = 1.04 [0.99–1.10], n = 3) [26,64,65,68]. See Figure 1 for forest plot.

Regarding secondary outcomes, two studies provided evidence on the association between physical maltreatment and measures of pain interference [26,64], both yielding null results (ns).

##### Verbal Abuse

One study [68] with 5001 participants yielded very low-quality evidence on the relationship between verbal abuse and pain at follow-up. Results adjusted for potential confounders showed a significant relationship between childhood verbal abuse and pain at follow-up; specifically, that higher frequency verbal abuse subsequently predicted an increased risk of pain (*p* < 0.001).

This study provided no evidence regarding secondary outcomes.

##### Neglect

Three studies [26,63,64] yielding very low-quality evidence from 1538 participants, provided information about the relationship between child neglect and pain at follow-up. In unadjusted analyses, Biskin and colleagues [63] reported a significant and positive association, such that more severe neglect was related to higher pain at follow-up (*p* < 0.01). However, adjusted findings from three studies [26,63,64] showed no significant relationship between history of neglect and pain at follow-up (ns).

In regard to secondary outcomes, adjusted analyses showed no association between neglect and measures of pain interference [26,64].

##### Exposure to Domestic Partner Violence

There was no available evidence regarding the relationship between exposure to domestic partner violence and pain or pain interference.

##### Exposure to Additional Maltreatment among Witnesses of Domestic Partner Violence

Lamers-Winkelman and colleagues [32] examined whether reports of maltreatment (abuse and neglect) were related to an increased risk for subsequent pain among children who had witnessed domestic partner abuse. The findings revealed that, compared to witnesses only, witnesses who had also been exposed to physical abuse were at increased risk for frequent stomach aches (*p* < 0.05) (but not headaches or aches and pains (ns)). When comparing witnesses only with witnesses who had also experienced any other form of abuse (emotional abuse, sexual abuse, and/or neglect, with or without physical abuse), significant differences in pain outcomes were not found (ns). There was no evidence regarding secondary outcomes.

#### 3.3.4. Does the Presence of Physical Injury Influence the Maltreatment–Pain Relationship?

Physical harm was coded as present in seven studies, including one study that measured the presence of penetration [69] and six studies that measured physical maltreatment [26,32,62,64,65,68]. There was no usable information reported by Rimsza et al. [69] on the relationship between penetration (yes/no) and pain. The remaining studies provided no additional evidence beyond what was discussed above regarding the association between physical maltreatment and pain.

#### 3.3.5. Does the Frequency and/or Chronicity of Maltreatment Influence the Relationship between Maltreatment and Pain?

There was insufficient evidence to perform a synthesis of whether maltreatment frequency or chronicity was related to pain outcomes. None of the studies provided usable information about the relationship between “frequent” maltreatment and pain, and only one study provided usable information about the relationship between “chronic” maltreatment (i.e., chronic neglect and chronic verbal and/or physical abuse) and pain [63]. Therefore, there was not enough evidence to examine whether the maltreatment–pain relationship depended on the intensity of the maltreatment—see Table S6 for GRADE summary.

#### 3.3.6. Does the Developmental Stage of Child Maltreatment Influence the Maltreatment-Pain Relationship?

Not one of the studies we included evaluated whether the developmental timing of maltreatment related to the maltreatment—pain relationship. The included studies did, however, capture maltreatment at different stages of development, with two studies that focused on maltreatment that occurred in early and middle childhood [26,32] and one study that measured exposure that occurred in adolescence [62]. However, because there were so few studies, we could not analyze whether developmental stage at the time of the exposure (i.e., maltreatment occurring in childhood vs. adolescence) related to later pain.

## 4. Discussion

The present review aimed to provide evidence on the longitudinal relationship between child maltreatment and pain. We aimed to delineate the specific nature of the maltreatment-pain relationship by examining potential moderating factors, including PTSD or PTSS, type of maltreatment, bodily injury, intensity of maltreatment (chronicity and frequency), and the developmental stage during which maltreatment occurred. Although the review included nine studies with data from 17,340 participants, the available evidence was limited across our research questions. In general, we found conflicting evidence regarding the relationship between child maltreatment and pain at follow-up, with the higher quality, well-adjusted studies more likely to reveal evidence of no association. Evidence from studies that accounted for the role of PTSD or PTSS suggests that psychological and emotional factors are key aspects of this phenomenon and may be essential to clarifying the nature of the maltreatment–pain relationship. However, only three of the nine studies we included measured chronic pain, our primary outcome variable. The remaining studies included in the review either did not specify pain duration or evaluated participants with acute pain (i.e., pain of ≤3 months’ duration). Consequently, the extent to which the present results apply to chronic pain is not clear.

### 4.1. Primary Findings

Findings from two low-risk-of-bias studies [26,62] point to a key role of PTSD/PTSS in the final statistical models suggesting that a complete model of maltreatment and pain must account for PTSD/PTSS. Consistent with our hypothesis, the Raphael and Widom [26] findings provided low-quality evidence that the nature of the maltreatment–pain relationship depended on the presence or absence of PTSD. Specifically, it was the combination of a maltreatment history and PTSD that was associated with risk of pain in adulthood, whereas neither maltreatment nor PTSD alone showed a robust association with pain. The replication of this finding with additional high-quality studies (i.e., studies with low risk of bias) is needed to increase our understanding of, and confidence, in this moderation effect.

In regard to the role of maltreatment type (sexual, physical, and emotion or verbal abuse, neglect, and exposure to domestic partner violence), there was low to very low-quality evidence was derived from zero to five studies examining the relationship between each type of maltreatment and pain at follow up. The pattern of results across studies showed some differentiation across abuse types. In particular, there was limited evidence (one study; very low quality) of an association between verbal abuse pain at follow-up, independent of baseline pain, other types of abuse, and other covariates. In contrast, the findings for physical abuse and pain (four studies; low quality evidence) and neglect and pain (three studies; low quality evidence) consistently showed no associations in models adjusted for covariates. Regarding sexual abuse, the evidence was conflicting, with inconsistent results across five studies (very low-quality evidence), regardless of whether or not the analyses were adjusted. Finally, there was no evidence regarding exposure to domestic partner violence and pain at follow up.

Despite some variation in the pattern of findings across abuse types, the most consistent (negative) findings are for physical abuse and neglect, in that they largely showed evidence of no association with pain at follow-up. Therefore, the findings on specific maltreatment types again point to a lack of a direct (non-moderated or non-mediated) association between maltreatment and pain, especially in adjusted models and when the exposure involves physical abuse or neglect. This begs the question of whether information on PTSD/PTSS is needed to delineate relationships of specific types of maltreatment to pain. Indeed, Raphael and Widom [26] showed evidence of moderation by PTSD across maltreatment types. These results provide additional evidence for the key role of PTSD in the maltreatment–pain relation and point to a lack of specificity when comparing sexual abuse, physical abuse, and neglect (the three types of abuse captured in the Raphael and Widom study) [26].

Unfortunately, we were not able to examine the roles of other proposed moderators (bodily injury, frequency of maltreatment, chronicity of maltreatment, and developmental stage) because the evidence was not available or if it was, it derived from only a single study, or there was not enough variability across studies to perform a between-study comparison.

### 4.2. Findings for Secondary Outcomes

In regard to secondary outcomes, four studies measured pain interference [26,63,64,66], and zero studies measured pain medication use. The findings for pain interference largely mirrored those of the primary results. Specifically, the evidence pointed to the absence of a direct association between maltreatment and pain interference, regardless of whether any maltreatment or a specific type of maltreatment (e.g., sexual abuse, physical abuse or neglect) was included in the model. Moreover, Raphael and Widom [26] reported the same interaction effect for pain interference as reported for pain, specifically, that it was the combination of child maltreatment and PTSD that was associated with increased pain interference in adulthood. Again, because this finding is based on a single study it should be interpreted with caution.

### 4.3. Additional Findings

Our review revealed some additional findings that, although pertinent to our research questions, provide only indirect evidence. First, the design of the Lamers-Winkelman et al. [32] study was unique in that it recruited child witnesses of domestic partner violence and then looked at the association between additional abuse exposure and parent-reported pain. Therefore, their models tested both the specificity of abuse types (e.g., physical abuse), in addition to the cumulative effect of exposure to multiple abuse types (i.e., witnessing violence plus experiencing physical abuse compared to witnessing violence alone). Results from this study generally showed no added risk of pain with the addition of physical abuse and other abuse types among child witnesses to domestic partner violence. Although this finding is based on a single study with high risk of bias, it provides some initial evidence that exposure to additional abuse types does not have a cumulative influence on pain outcomes, a finding that is inconsistent with the cumulative effects shown across the Adverse Child Experiences (ACEs) literature [19,22].

Second, given the reported findings on mediation by PTSS [62], and the fact that mediation models shed light on our research question about the prospective relationship been maltreatment and pain, we discuss these findings in the paragraphs below. However, because these results were based on post hoc tests and were not subject to our quality appraisal, they should be assumed to be based on very low quality evidence. As described above, Beal et al. [62] showed that PTSS fully mediated the maltreatment-pain relationship, indicating that PTSS is more proximally related to pain. Evidence from other studies in the review also points to PTSD/PTSS as an important pathway. Specifically, Sachs-Ericsson and colleagues [68] reported findings consistent with a mediation model, such that the significant associations between maltreatment indices (verbal abuse and sexual abuse) and pain at follow-up were not maintained when PTSD was added to the model. Finally, Kopec and Sayre [65] also reported findings consistent with a mediation model. These authors reported a high degree of correlation between physical abuse and a measure capturing the experience of significant fear in childhood (i.e., “Did something happened that scared you so much you thought about it for years after?” p. 479) with 68% of respondents who reported physical abuse also reporting fear. In statistical models that included both childhood physical abuse and fear, only the latter remained significantly associated with pain. This finding provides further evidence that the emotional response to maltreatment may be at the core of the maltreatment–pain relationship. That said, not all studies that measured PTSD and related indices found evidence of consistent mediation. Specifically, Raphael and Widom [26] reported that there was no evidence of mediation by PTSD (although it should be noted that the results of the statistical analysis for mediation were not fully reported in the published paper), and Biskin and colleagues [63] reported that their results failed to show an association between baseline PTSD and pain at follow-up, a finding that is also inconsistent with mediation by PTSD.

### 4.4. Strengths and Limitations of the Review

This review has several strengths. For one, we planned the methods in advance and registered the study with PROSPERO before we performed the literature search. This included specifying a set of potential moderating variables associated with the presence of PTSD or PTSS, the specific nature of child maltreatment (i.e., its type and intensity and whether it involved physical harm), and other factors possibly related to heterogeneity, including whether pain was measured in childhood or adulthood. Similarly, we defined, in advance, the requirements for a minimally versus adequately adjusted statistical model and accounted for this when assessing of risk of bias. Secondly, to identify studies to include in the present review, we conducted a comprehensive electronic search of the literature followed by a review of the reference sections of influential original articles and reviews. Thirdly, we accounted for risk of bias, effect size and precision of effect, heterogeneity, generalizability, and potential reporting bias when judging the overall quality of the evidence.

The present review also has limitations. The main limitation relates to the dearth of evidence in combination with the variability among the studies in the measurement of maltreatment and pain, which rendered cross-study comparisons largely inconclusive. For example, some studies specified contact sexual abuse (e.g., rape or molestation) in their definitions of sexual abuse, e.g., [68], whereas Linton et al. [66] included both contact abuse and non-contact abuse (someone exposing their sex organs), thereby capturing sexual abuse at a different threshold and finding different results. As such, it is unclear whether inconsistent findings across studies (e.g., the significant association reported by Sachs-Ericsson and colleagues [68] versus the absence of an association reported by Linton and colleagues [66]) can be attributed to varying definitions of sexual abuse, different operationalizations and measurement of pain, or variation in the specific covariates included in the statistical models.

An additional limitation is that the present review likely does not include the entirety of research on the topic. Given that we conducted a prognostic factor review, we expect there are reporting and publication biases given the challenges associated with publishing studies that fail to show a relationship between maltreatment and pain. Additionally, we only included English language publications, which very likely missed a pool of published studies. Another limitation is the relatively poor internal validity (risk of bias) of included studies, given the potential influence of confounding factors (baseline pain, current/adult abuse, and co-occurring abuse types). Finally, a limitation of the literature included in the present review is that all studies are observational and, therefore, causality cannot be implied or inferred from their results. Thus, although the higher quality, well-adjusted studies tended to reveal evidence of no association between maltreatment and subsequent pain, when lower quality studies showed such an association, there is no evidence or suggestion that maltreatment causes pain.

### 4.5. Agreements and Disagreement with Other Reviews

Compared to the findings presented in the current review, reviews on similar topics published over the last decade have generally pointed to a more consistent and robust relationship between child maltreatment and pain and pain-related outcomes across maltreatment types. Indeed, reviews by Afari et al. [53], Häuser et al. [54], and Paras et al. [8], each reported large effect sizes, indicating increased risk of pain and somatic disorders among participants who reported a history of maltreatment. Although we can only speculate on the reasons for this discrepancy, it may be a reflection of the quality of evidence in our review versus the earlier reviews, with the latter suffering from higher risk of bias.

In an effort to illuminate the temporal nature of the maltreatment–pain relationship and bolster the overall quality of the evidence, we limited the present review to studies that used prospective measures of child maltreatment. In contrast, the aforementioned reviews cast a wider net by also including case–control studies, and as a result, the vast majority of evidence is from studies of this design. In particular, the Häuser et al. [54] review of sexual abuse and fibromyalgia syndrome was based on 18 case–control studies, the Paras et al. [8] review of sexual abuse and somatic disorders (including non-specific chronic pain and chronic pelvic pain) was based on 19 case–control studies and four cohort studies, and the Afari et al. [53] review of psychological trauma (including child maltreatment) and somatic conditions (such as chronic widespread pain and fibromyalgia) was based on 58 case–control studies and 13 cohort studies. Each of these reviews included meta-analyses and reported impressive effects, including the Paras et al. [8] finding that individuals with a sexual abuse history were 2.2 times more likely to receive a diagnosis of non-specific chronic pain and the Afari et al. [53] finding that individuals with a history of emotional abuse were 2.11 times more likely to have a somatic condition. The problem is that case–control studies are subject to high risk of bias, including both participation/sampling and measurement bias, thereby lowering our confidence in the combined effects reported.

Early reviews on the topic of child maltreatment and chronic pain identified many of the same methodological concerns discussed above [11,14]. Fifteen years later, we found only nine studies examining the prospective association between child maltreatment and pain. Although we consider these nine studies to reflect the best available evidence on this topic, our GRADE analysis revealed designations of “low quality”, “very low quality” and “no available evidence” across our research questions, reflecting the significant methodological challenges faced by the field. High quality studies continue to be published, as exemplified by the Beal et al. [62] study included in the current review, albeit the progress is slow. In the meantime, creative solutions are needed to address key methodological concerns in a timelier way, a topic that we turn to in the paragraphs below.

### 4.6. Implications for Research

The results of the present review suggest that there is no direct association between maltreatment and pain, but that PTSD or symptoms of PTSD may play a role as a mediator and/or a moderator of this relationship. However, our findings are based on low- to very low-quality evidence; therefore, further high quality studies are required to characterize the nature of this association. This is important because reviews of case–control studies point to a robust relationship between child maltreatment and pain [8,54], and the literature on ACEs suggests that child maltreatment is one of a number of childhood adversities that has a dose-effect relationship with pain outcomes [19,22]. Therefore, if child maltreatment is on its own not a risk factor for later pain, this will have important implications for future research and prevention efforts. On the other hand, if child maltreatment only acts in combination with PTSD or at high levels of intensity (i.e., frequent exposures over prolonged periods), this is equally critical for guiding future work.

Research in this area would benefit from large, well-controlled prospective cohort studies using decade-long follow ups to examine the temporal relationship between child maltreatment and chronic pain, as well as formal tests of mediation and moderation by PTSD and other theoretically derived variables (such as the moderators proposed in this review). However, given the significant amount of time and additional resources needed to obtain such data, we recommend applying novel methods to accelerate research in this field. One possibility would be to study the transition from acute, time-limited pain to chronic pain. For example, Salberg et al. [72] have proposed surgical procedures and traumatic brain injury (both of which increased risk for chronic pain) as models for investigating the transition from acute to chronic pain in adolescence. In this regard, child maltreatment could be examined as a risk factor for the emergence of chronic pain following these events. Another possibility would be to take a life course perspective by collecting detailed maltreatment histories (including early life, repeated, and/or current exposure) [20,73]. Trajectories of maltreatment could then be examined in relation to the emergence of pain across critical stages, such over the course of adolescence [74].

Given that it is often necessary to measure maltreatment retrospectively, to reduce the impact of reporting biases and increase the validity of these measures, we recommend the use of standardized measures with multiple informants (self-, sibling-, and parent-reports) in combination with official records (e.g., court documentation) [20]. The aim should be to capture specific types of maltreatment and co-occurring maltreatment types, including the frequency and chronicity of these exposures over time [20,73]. We also recommend that future work incorporate formal tests of mediation and moderation by PTSD and symptoms of PTSD, including in-depth analysis of PTSD symptom clusters of re-experiencing, avoidance, and arousal, e.g., [75,76]. In this regard, it will also be important to delineate the characteristics of child maltreatment that increase the likelihood of the emergence of PTSD or symptoms of PTSD and chronic pain, such as the presence of rape in child sexual abuse [8,31], or more generally, the presence of physical injury [30]. As part of this, future work should determine whether the presence or absence of a clinical diagnosis of PTSD explains variance in pain outcomes over and above symptoms of PTSD (as measured on a continuous scale).

In addition to in-depth measurements of child maltreatment, we recommend comprehensive pain assessment, including the use of well-validated and reliable measures that provide information about pain severity, location, and pain interference (e.g., Brief Pain Inventory-Short Form (BPI-SF)) [77,78], as well as pain location and quality (e.g., Short Form-McGill Pain Questionnaire (SF-MPQ)) [79]. These measures are typically used with adults, but they can also be used with children and adolescents [80]. Finally, we recommend repeated symptom measures to capture changes in pain outcomes over time, as well as dynamic associations between symptoms of pain and symptoms of PTSD across development [17,76].

## Supplementary Materials

The following are available online at https://www.mdpi.com/article/10.3390/children8090806/s1. References [81,82,83,84,85,86,87,88,89,90,91] are cited in the Supplementary Materials.
