# Supplementary material for: A Systematic Review of the Prospective Relationship between Child Maltreatment and Chronic Pain
Source: Children (Basel). 2021 Sep 15;8(9):806. doi: 10.3390/children8090806 (PMC8469564; doi:10.3390/children8090806)
Supplement: Supplementary file 1 [file children-08-00806-s001.zip › children-1318780-supplementary.pdf]

children

MDPI

Supplementary Materials

Table S1. *Coding of Maltreatment-Related Characteristics*

Characteristic of Child Maltreatment	Definition
Type	We coded for the kind of child maltreatment, specifically physical abuse, sexual abuse, neglect, emotional maltreatment, and domestic partner violence exposure. For sexual abuse we coded for the presence of penetration (vaginal, anal, or oral) with a body part or foreign object. We also coded for the presence of physical violence (yes/no). We considered physical violence to be present in cases of physical abuse, rape, and other violent sexual acts. When different types of maltreatment were combined in a single measure, we coded the type of maltreatment as unspecified. Given that various kinds of abuse often occur together [20], we noted whether or not this was accounted for in the measurement of maltreatment and/or using statistical controls.
Bodily harm	We coded for bodily harm resulting from physical and sexual abuse (yes/no). We also recorded information pertaining to the specific type of harm or injury (e.g., laceration, burn, fracture). Finally, we recorded any additional information about the occurrence of other types of childhood injuries (e.g., accidental injury and intentional self-injury) because of their associations with child maltreatment [32,34,35].
Frequency	Frequent maltreatment was defined as maltreatment that occurred more than 10 times or was rated as occurring "often" or "frequently." This definition is based on frequency data reported by Anderson and colleagues [91] indicating that among women who had been sexually abused, 58% of whom reported a single instance of abuse, 28% reported having been abused 2-10 times, and 14% more than 10 times. Therefore, our definition captures the higher intensity of maltreatment experienced by a portion of survivors
Chronicity	Chronic child maltreatment was defined as maltreatment that persisted for at least six months or was reported to be present at two measurement points spaced at least six months apart. This definition is consistent with definitions of chronic stress, where the stressor needs to be present for at least six months [92].
Developmental Stage	We defined early childhood as 0-5 years of age, middle childhood as 6 to 10 years of age, and adolescence as 11 to 18 years of age [23,24,43].

Table S2. Search Terms and Yields for Review of Traumatic Events and Chronic Pain

Database: Epub Ahead of Print, In-Process and Other Non-Indexed Citations, Ovid MEDLINE(R) Daily and Ovid MEDLINE(R) <1946 to Present>

Search Strategy:

----

1 (adverse adj3 event?).ti,ab. (133405)

2 (adverse adj3 incident?).ti,ab. (556)

3 Stress Disorders, Post-Traumatic/ (28447)

4 stress disorders, traumatic/ or battered child syndrome/ or combat disorders/ or stress disorders, traumatic, acute/ (4799)

5 post-traumatic stress.ti,ab. (9750)

6 posttraumatic stress.ti,ab. (17510)

7 PTSD.ti,ab. (19269)

8 (severe adj3 accident$).ti,ab. (864)

9 (severe adj3 injur$).ti,ab. (27141)

10 Survivors/px [Psychology] (8397)

11 trauma$.ti,ab. (310084)

12 Bullying/ (2376)

13 Child Abuse/ (20906)

14 Child Abuse, Sexual/ (9644)

15 Rape/ (6197)

16 Sex Offenses/ (7656)

17 (abus$ adj3 (child or physical or sexual)).ti,ab. (23042)

18 or/1-17 (521868)

19 Chronic Pain/ (8151)

20 (chronic adj3 pain).ti,ab. (50977)

21 Chronic Disease/ (255754)

22 chronic condition?.ti,ab. (13894)

23 Pain/ and (recur$ or chronic or persistent).ti,ab. (22437)

24 (pain? adj5 (recur$ or chronic or persistent)).ti,ab. (69710)

25 Pain/ep, et, px [Epidemiology, Etiology, Psychology] (54311)

26 Pain, intractable/ (6144)

27 Fibromyalgia/ (7686)

28 Irritable Bowel Syndrome/ (6044)

29 Arthritis/ (35406)

30 exp back pain/ and (recur$ or chronic or persistent).ti,ab. (8981)

31 neck pain/ and (recur$ or chronic or persistent).ti,ab. (1542)

32 Osteoarthritis/ (35175)

33 Arthritis, Rheumatoid/ (94135)

34 Headache/ and (recur$ or chronic or persistent).ti,ab. (3966)

35 Migraine/ and (recur$ or chronic or persistent).ti,ab. (3746)

36 Neuralgia/ (11475)

37 Peripheral Nervous System Diseases/ (22216)

38 Complex Regional Pain Syndromes/ (1172)

39 Whiplash Injuries/ and (recur$ or chronic or persistent).ti,ab. (705)

40 Cumulative Trauma Disorders/ and (recur$ or chronic or persistent).ti,ab. (488)

41 Somatoform Disorders/ and (recur$ or chronic or persistent).ti,ab. (1549)

42 (somat$ adj3 pain).ti,ab. (1961)

43 or/19-42 (557592)

44 impact$.ti,ab. (834620)

45 Influen$.ti,ab. (1411837)

46 predict$.ti,ab. (1315420)

47 factor$.ti,ab. (2952954)

48 association?.ti,ab. (1128022)

49 Association/ (3869)

50 cross-sectional.ti,ab. (263712)

51 risk.mp. (2144345)

52 Risk/ (118699)

53 (related or relation$).ti,ab. (3392028)

54 correlat$.ti,ab. (1649023)

55 aggregat$.ti,ab. (215239)

56 or/44-55 (9704939)

57 18 and 43 and 56 (11035)

58 animals/ not humans/ (4636441)

59 57 not 58 (10634)

Table S3. Variables extracted from articles included in the review.

**Variable information extracted**
Patient characteristics
Sample size
Study setting
Follow-up period
Child maltreatment (yes/no)
Type of child maltreatment (physical abuse, emotional abuse, sexual abuse, neglect, and exposure to domestic partner violence)
Additional characteristics of child maltreatment, including the presence of physical violence (yes/no) and penetrative sexual assault (yes/no)
Co-occurrence of different types of child maltreatment
Frequency and chronicity of child maltreatment
Developmental stage during which maltreatment occurred
Presence and type of abuse-related physical harm
Presence of additional childhood physical harm, including non-inflicted injury and self-harm (yes/no)
Post-traumatic stress disorder (yes/no)
Post-traumatic stress symptoms
Presence of current abuse or adult abuse (yes/no)
Pain at baseline
Pain outcome measures used to determine pain intensity or incidence
Presence of a chronic pain condition

Figure S1. *Study Flow Diagram.*

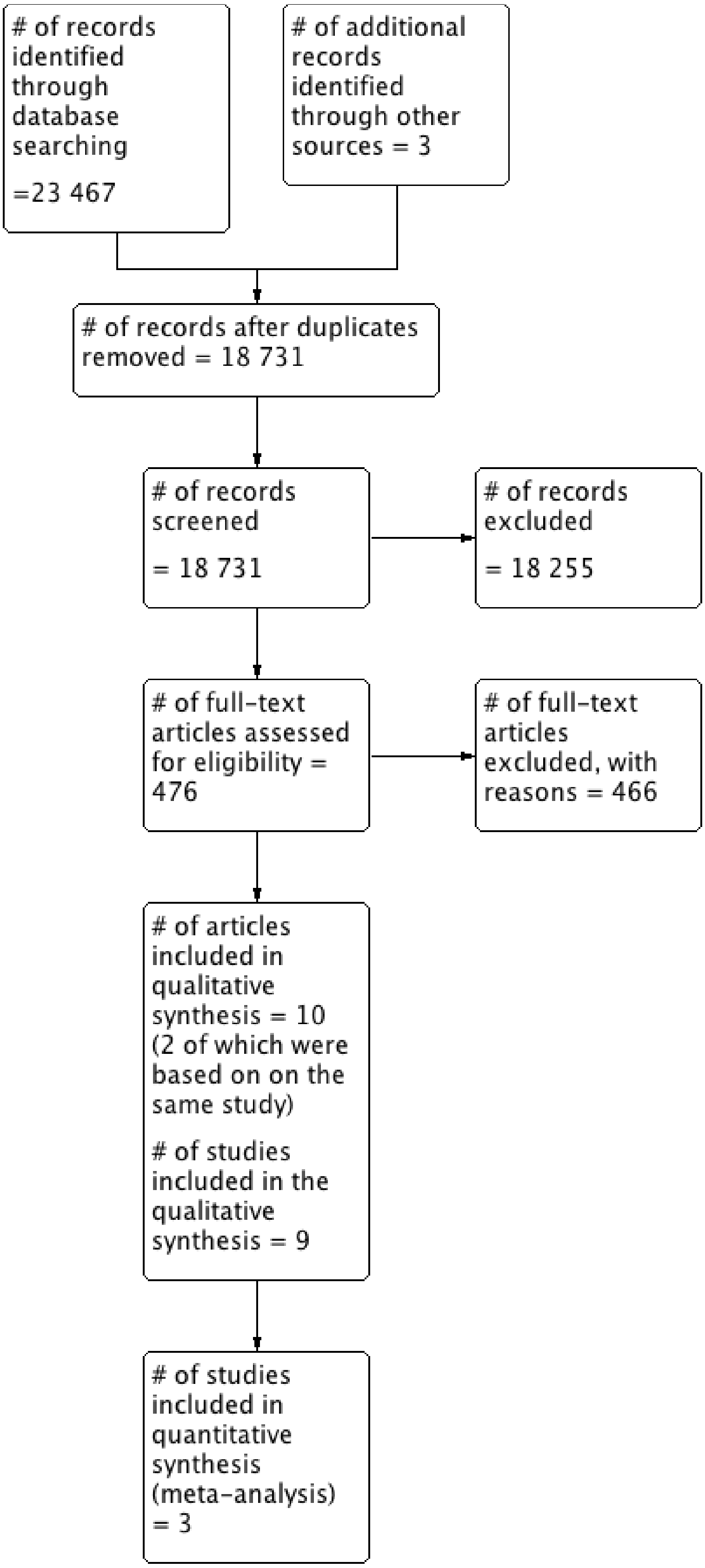

children

MDPI

Table S4. Characteristics of included studies.

	Number of subjects with complete data	Age at baseline (mean (SD) or range in years) plus baseline pain status	Child maltreatment measure plus prevalence data	Developmental timing of maltreatment	Recency of maltreatment exposure	Measurement, reporting, and analysis of proposed moderators	Measures of pain and pain interference, and whether chronic pain was captured	Pain measurement timepoint(s)[1]
Beal 2020	383	Maltreated<br>15.2 (1.1)<br><br>Comparison<br>15.3 (1.04)<br><br>Caregiver reports of pain at baseline = 49% in the maltreatment and 59% in the comparison sample for past 2-year pain symptoms	Official reports + structured interview<br><br>Comprehensive Trauma Interview for maltreatment experiences by type [70].<br><br>Of 273 cases, there were 31% allegations for physical abuse, 49% for sexual abuse, 15% for	Adolescence	Approximately 10 years	Moderators measured<br>Abuse type (sexual abuse, physical abuse, neglect)<br>PTSS (Comprehensive Trauma Interview) [70]<br>Moderators reported[2]<br>Abuse type (abuse only, neglect only, both abuse and neglect)<br>PTSS	Measure of pain = self-reported pain (yes/no)<br>Measure of pain interference? No<br><br>Brief Pain Inventory [77]<br><br>Chronic pain captured? No	Baseline and 9-year follow-up

	**Number of subjects with complete data**	**Age at baseline (mean (SD) or range in years) plus baseline pain status**	**Child maltreatment measure plus prevalence data**	**Developmental timing of maltreatment**	**Recency of maltreatment exposure**	**Measurement, reporting, and analysis of proposed moderators**	**Measures of pain and pain interference, and whether chronic pain was captured**	**Pain measurement timepoint(s)[1]**
			neglect, 5% for multiple types			Moderators analyzed[3]<br>None		
Biskin 2014	231	27.0 (6.3)<br><br>Pain at baseline in borderline personality disorder sample = unknown	Semi-Structured Interview<br><br>Revised Childhood Experiences Questionnaire (CEQ-R) [81]<br><br>No descriptive data provided regarding maltreatment exposures in BPD sample	Unknown (<18 years)	Unknown	Moderators measured<br>Abuse type (sexual (yes/no), verbal and physical combined, neglect)<br>Chronic maltreatment<br>PTSD<br>The Structured Clinical Interview for DSM-III-R Axis I Disorders (SCID-I) [82]<br>Moderators reported	Measure of pain = self-reported pain severity<br>Measure of pain interference? Yes<br><br>The Brief Pain Inventory [77]<br><br>Chronic pain captured? No	16-year follow-up

	**Number of subjects with complete data**	**Age at baseline (mean (SD) or range in years) plus baseline pain status**	**Child maltreatment measure plus prevalence data**	**Developmental timing of maltreatment**	**Recency of maltreatment exposure**	**Measurement, reporting, and analysis of proposed moderators**	**Measures of pain and pain interference, and whether chronic pain was captured**	**Pain measurement timepoint(s)[1]**
						Abuse type (sexual (yes/no), verbal and physical combined, neglect)<br>Chronic maltreatment<br>PTSD<br><u>Moderators analyzed</u><br>None		
Brown 2005	602	22<br><br>Pain at baseline: 13.4% of sample reported frequent pain and 18.5% of sample reported chronic	Official reports<br><br>4.6% of sample had a documented history of physical abuse and/or neglect, including	Unspecific (<18 y.o.)	Unknown	<u>Moderators measured</u><br>Abuse type (sexual abuse, physical abuse, neglect)<br><u>Moderators reported</u>	Measure of pain = self-reported “frequent pain”<br>Measure of pain interference? Yes<br><br>Measured using a single	All data collected at single timepoint

	**Number of subjects with complete data**	**Age at baseline (mean (SD) or range in years) plus baseline pain status**	**Child maltreatment measure plus prevalence data**	**Developmental timing of maltreatment**	**Recency of maltreatment exposure**	**Measurement, reporting, and analysis of proposed moderators**	**Measures of pain and pain interference, and whether chronic pain was captured**	**Pain measurement timepoint(s)[1]**
		pain in past year that impaired functioning.	14 cases of physical abuse, 16 cases of neglect (with no abuse), and 4 cases of sexual abuse.			Abuse type (physical abuse, neglect)<br>*Too few cases of sexual abuse to examine separately<br><u>Moderators analyzed</u><br>None	question for each.<br><br>Chronic pain captured? Yes, but for pain impairment only	
Kopec 2005	9552	Age 18-24 11.8%<br>Age 25-44 41%<br>Age 45-64 26.8%<br>Age 65+ 20.4%	Self-report questionnaire<br><br>Single question developed by researchers at Statistics Canada based on unpublished analyses.	Unspecified (<18 y.o.)	Unknown	<u>Moderators measured</u><br>Abuse type (physical)<br><u>Moderators reported</u><br>Abuse type (physical)<br><u>Moderators analyzed</u><br>None	Measure of pain = self-reported onset of back pain<br>Measure of pain interference? No<br><br>Interviewer-administered questionnaire	Baseline and 4-year follow-up

	**Number of subjects with complete data**	**Age at baseline (mean (SD) or range in years) plus baseline pain status**	**Child maltreatment measure plus prevalence data**	**Developmental timing of maltreatment**	**Recency of maltreatment exposure**	**Measurement, reporting, and analysis of proposed moderators**	**Measures of pain and pain interference, and whether chronic pain was captured**	**Pain measurement timepoint(s)[1]**
		Pain at baseline: Participants were free of back pain at baseline.	6.5% of sample reported any exposure to physical abuse				(single question).<br><br>Chronic pain captured? Yes	
Lamers -Winkelman 2012	275	8.62 (1.7)<br><br>Pain at baseline: 7.7% aches and pains, 9.9% headache, and 13.5% stomach ache among children exposed to domestic partner violence	Maltreatment data collected from multiple informants<br><br>Parent reports (Parent Report of Traumatic Impact [83] plus interviews)<br>Official agency reports	Middle childhood	Unknown	<u>Moderators measured</u><br>Abuse type (physical abuse, contact sexual abuse, emotional abuse (recurrent humiliation), neglect)<br><u>Moderators reported</u><br>Abuse type (physical abuse, contact sexual abuse, emotional	Measure of pain = parent reports of aches and pains, stomach ache, headache<br>Measure of pain interference? No<br><br>Somatic Complaints Scale within the	All data collected at single timepoint

	**Number of subjects with complete data**	**Age at baseline (mean (SD) or range in years) plus baseline pain status**	**Child maltreatment measure plus prevalence data**	**Developmental timing of maltreatment**	**Recency of maltreatment exposure**	**Measurement, reporting, and analysis of proposed moderators**	**Measures of pain and pain interference, and whether chronic pain was captured**	**Pain measurement timepoint(s)[1]**
			Prevalence of additional maltreatment among children exposed to domestic partner violence: 53% for physical abuse, 11% for sexual abuse, 36% for emotional abuse, and 36% for neglect			abuse (recurrent humiliation), neglect)<br><u>Moderators analyzed</u><br>None	Child Behavior Checklist [84]<br><br>Chronic pain captured? Yes	
Linton 2002	417	35 – 45<br><br>Pain at baseline:<br><u>No pain group</u> (46%): participants reported	Self-report questionnaire<br><br>5 questions from previous studies [85,86]	Unspecified (<14 y.o.)	>20 years	<u>Moderators measured</u><br>Abuse type (sexual)<br>Frequent sexual abuse	Measure of pain = self-reported spinal pain. Measure of pain interference? Yes	Baseline and one-year follow-up

	Number of subjects with complete data	Age at baseline (mean (SD) or range in years) plus baseline pain status	Child maltreatment measure plus prevalence data	Developmental timing of maltreatment	Recency of maltreatment exposure	Measurement, reporting, and analysis of proposed moderators	Measures of pain and pain interference, and whether chronic pain was captured	Pain measurement timepoint(s)[1]
		that they had not suffered any spinal pain in past year<br>Pain group (54%)—any pain in the past year	Prevalence data includes adult exposures—23% reported some form of sexual abuse and 22% reported physical abuse			Moderators reported<br>Abuse type (sexual)<br>Moderators analyzed<br>None	Pain was measured using questions to assess back and neck pain, and if present, pain intensity. Physical function was measured using self- administered physical function tests [87].<br><br>Chronic pain captured? Unclear for pain and no for physical function.	

	**Number of subjects with complete data**	**Age at baseline (mean (SD) or range in years) plus baseline pain status**	**Child maltreatment measure plus prevalence data**	**Developmental timing of maltreatment**	**Recency of maltreatment exposure**	**Measurement, reporting, and analysis of proposed moderators**	**Measures of pain and pain interference, and whether chronic pain was captured**	**Pain measurement timepoint(s)[1]**
Raphael 2011 (and Raphael 2001)	807	29.1 years<br><br>Pain at baseline: pain symptom counts for any maltreatment group was 2.51 (0.08) and for control group was 2.32 (0.09)	Official reports<br><br>Of 458 with documented maltreatment, 13% cases of sexual abuse, 17% cases of physical, and 81% cases of neglect	Early childhood and middle childhood	Approximately 30 years	Moderators measured<br>Abuse type (physical abuse, sexual abuse, and neglect)<br>PTSD (National Institutes of Mental Health Diagnostic Interview Schedule Version III-R) [47].<br>Moderators reported<br>Abuse type (physical abuse, sexual abuse, and neglect)<br>PTSD	Measure of pain = self-reported pain symptoms<br>Measure of pain interference? Yes<br><br>All pain indices derived from the somatization module of the Diagnostic Interview Schedule III-R [47]<br><br>Chronic pain captured? No	Baseline and 10-year follow-up

	**Number of subjects with complete data**	**Age at baseline (mean (SD) or range in years) plus baseline pain status**	**Child maltreatment measure plus prevalence data**	**Developmental timing of maltreatment**	**Recency of maltreatment exposure**	**Measurement, reporting, and analysis of proposed moderators**	**Measures of pain and pain interference, and whether chronic pain was captured**	**Pain measurement timepoint(s)[1]**
						<u>Moderators analyzed</u><br>PTSD		
Rimsza 1988	144	10 (with range of 2-17)<br><br>Pain at baseline = unknown	Clinical interview, with verification by physical examination for patients aged 2-4.<br><br>Of 72 sexual abuse patients, 61% reported penile vaginal penetration, 28% reported genital fondling, including nonpenile	Early childhood, middle childhood, and adolescence	Unknown (although it appears that exposures were quite recent)	<u>Moderators measured</u><br>Abuse type (penile vaginal penetration, no penile vaginal penetration)<br>Stable/prolonged abuse<br><u>Moderators reported</u><br>Abuse type (penile vaginal penetration, no penile vaginal penetration)	Measure of pain = headaches, chest pain, back pain, abdominal pain, vaginal pain<br>Measure of pain interference? No<br><br>Measured via chart review, sometimes with the addition of phone interview with	2-year follow-up (with range from 9 to 48 months)

	Number of subjects with complete data	Age at baseline (mean (SD) or range in years) plus baseline pain status	Child maltreatment measure plus prevalence data	Developmental timing of maltreatment	Recency of maltreatment exposure	Measurement, reporting, and analysis of proposed moderators	Measures of pain and pain interference, and whether chronic pain was captured	Pain measurement timepoint(s)[1]
			vaginal penetration, 12.5% reported sodomy, 19.4% reported oral-genital contact (categories are not mutually exclusive).			Stable/prolonged abuse<br>Moderators analyzed<br>None	primary caregiver.<br><br>Chronic pain captured? Unclear for all outcomes	
Sachs-Ericsson 2017	5001	33.03 (10.5)<br><br>Pain at baseline -Pain experienced as a result of health problems (scale ranging from 0 to 3)—mean baseline scores	Semi-structured interview<br><br>Items from the PTSD module of the Composite International Diagnostic Interview [89] and the	Unspecified (<18 y.o. for verbal and physical abuse and neglect and <15 y.o. for sexual abuse)	30 - 40 years	Moderators measured<br>Abuse type (sexual, physical, emotional/verbal)<br>Frequent abuse<br>PTSD (Composite International Diagnostic Interview) [89]	Measure of pain = self-reported number of painful medical disorders (arthritis or rheumatism, chronic back or neck problems, fre-	Baseline pain severity<br>Painful medical disorders from<br>10-year follow-up

	**Number of subjects with complete data**	**Age at baseline (mean (SD) or range in years) plus baseline pain status**	**Child maltreatment measure plus prevalence data**	**Developmental timing of maltreatment**	**Recency of maltreatment exposure**	**Measurement, reporting, and analysis of proposed moderators**	**Measures of pain and pain interference, and whether chronic pain was captured**	**Pain measurement timepoint(s)[1]**
		was 0.3 (SD=.77)	childhood history section of the National Comorbidity Survey (NCS-1)<br><br>4.1% of participants reported physical abuse that occurred "sometimes" or "often," 28.6% reported verbal abuse that occurred sometimes or often, and 2.9% reported sexual abuse that occurred more than once.			<u>Moderators reported</u><br>Abuse type (sexual, physical, emotional/verbal)<br>Frequent abuse<br>PTSD<br><u>Moderators analysed</u><br>None	quent or severe headache, or other chronic pain)<br>Measure of pain interference? No<br><br>Questions from the National Comorbidity Survey [90]<br><br>Chronic pain captured? Unclear	

Notes

[1]Given that some studies relied on retrospective reports of maltreatment, the timespan between the maltreatment exposure and pain measurement often exceeds that of study follow-up period.

[2]Indicates whether descriptive information and/or the association between the moderator and pain outcome(s) was reported.

[3]Indicates whether a moderation analysis was conducted and reported.

Table S5. Summary of risk of bias assessment based on the Quality in Prognosis Studies tool [56]. Judgments (low, moderate or high) for the six risk-of-bias domains shown for each study and the overall study rating for each (low or high).

Study	Study participation	Study attrition	Measurement of exposure	Measurement of outcome	Study confounding	Statistical analysis and	Overall rating
Beal 2020	Low	Low	Low	Low	Moderate	Low	Low
Biskin 2014	Moderate	Moderate	Moderate	Low	Moderate	High	High
Brown 2005	Low	Low	Low	Moderate	Moderate	Low	Low
Kopec 2005	Low	Low	Moderate	Moderate	Moderate	Low	Low
Lamers-Winkelman 2012[a]	Moderate	N/A	Moderate	Low	High	High	High
Linton 2002[b]	Low	Low	Moderate	Moderate / Low	Moderate	Low	Low
Raphael 2011	Low	Moderate	Low	Low	Moderate	Moderate	Low
Rimsza 1988	Moderate	High	Low	High	Moderate	High	High
Sachs-Ericsson 2017	Low	Low	Moderate	Moderate	Moderate	Low	Low

● (green) Low Risk of Bias

● (grey) Moderate Risk of Bias

● (red) High Risk of Bias

Notes.

[a]The Lamers-Winkelman et al. (2012) study was not rated along the Study Attrition dimension because this was a cross-sectional study. Although the Brown et al. (2005) findings were also based on cross-sectional data, these data were from a larger cohort study, therefore study attrition remained a potential source of bias.

[b]Two risk-of-bias ratings are reported for the Measurement of Outcome domain because there were two reported outcomes with differing ratings (i.e., moderate for the measure of pain and low for the measure of pain interference). In cases of multiple outcomes reported in other studies, the risk of bias ratings was constant across outcomes.

Table S6. Grading of Recommendations, Assessment, Development and Evaluation (GRADE).

Research question	Exposure variable	Number of studies (participants)	Unadjusted			Adjusted			GRADE Factors										
			+	0	-	+	0	-	Number of studies (participants) in meta-analysis	Odds ratio (95% CI) for adjusted analysis	Study limitations	Inconsistency	Indirectness	Imprecision	Publication bias	Moderate/large effect size	Dose effect	Overall quality
What is the relationship between any child maltreatment and pain at follow-up?	**Any child maltreatment (sexual abuse, physical abuse, and/or neglect)**	3 (1,421)	2	0	0	1	2	0	0	NA	✓	✓	X	✓	X	X	X	++
What is the evidence regarding	**PTSD or PTSS**	1 (807)	0	0	0	1	0	0	0	NA	✓	NA	X	✓	X	✓	X	++

**Research question**	**Exposure variable**	**Number of studies (participants)**	**Unadjusted**			**Adjusted**			**Number of studies (participants) in meta-analysis**	**Odds ratio (95% CI) for adjusted analysis**	**GRADE Factors**							
			**+**	**0**	**-**	**+**	**0**	**-**			**Study limitations**	**Inconsistency**	**Indirectness**	**Imprecision**	**Publication bias**	**Moderate/large effect size**	**Dose effect**	**Overall quality**
moderating variables?	**Specific type of maltreatment**																	
	Sexual	5 (6,203)	1	1	0	1	2	0	0	NA	X	X	X	X	X	X	X	+
	Physical	4 (15,150)	0	0	0	0	4	0	3 (14,723)	1.04 (0.99-1.10)	✓	✓	X	✓	X	X	X	+ +
	Emotional/verbal	1 (5,001)	0	0	0	1	0	0	0	NA	✓	NA	X	✓	X	X	X	+
	Neglect	3 (1,538)	1	0	0	0	3	0	0	NA	✓	✓	X	✓	X	X	X	++
	Exposure to violence	N/A																No evidence
	**Presence of physical harm/injury**	N/A																No evidence
	**Frequent maltreatment**	N/A																No evidence
	**Chronic maltreatment**	N/A																No evidence

**Research question**	**Exposure variable**	**Number of studies (participants)**	**Unadjusted**			**Adjusted**			**GRADE Factors**									
			**+**	**0**	**-**	**+**	**0**	**-**	**Number of studies (participants) in meta-analysis**	**Odds ratio (95% CI) for adjusted analysis**	**Study limitations**	**Inconsistency**	**Indirectness**	**Imprecision**	**Publication bias**	**Moderate/large effect size**	**Dose effect**	**Overall quality**
	**Developmental stage of maltreatment**	N/A																No evidence

Note. For unadjusted and adjusted analyses: +, number of significant effects in expected direction; 0, number of non-significant effects; -, number of significant effects in unexpected direction. For GRADE factors, ✓, no serious limitations; X, serious limitations (or not present for moderate/large effect size, dose effect). For overall quality of evidence: +, very low; ++, low; +++, moderate; ++++, high.
